# The α7-nicotinic receptor is upregulated in immune cells from HIV-seropositive women: consequences to the cholinergic anti-inflammatory response

**DOI:** 10.1038/cti.2015.31

**Published:** 2015-12-11

**Authors:** Manuel Delgado-Vélez, Carlos A Báez-Pagán, Yamil Gerena, Orestes Quesada, Laura I Santiago-Pérez, Coral M Capó-Vélez, Valerie Wojna, Loyda Meléndez, Rosiris León-Rivera, Walter Silva, José A Lasalde-Dominicci

**Affiliations:** 1 Department of Biology, University of Puerto Rico, Río Piedras Campus, San Juan, Puerto Rico; 2 School of Medicine, Department of Pharmacology, University of Puerto Rico, San Juan, Puerto Rico; 3 Department of Physical Sciences, University of Puerto Rico, San Juan, Puerto Rico; 4 Specialized Neuroscience Research Program in Neuro AIDS, University of Puerto Rico, San Juan, Puerto Rico; 5 Internal Medicine, Neurology Division, University of Puerto Rico, San Juan, Puerto Rico; 6 Department of Microbiology and Medical Zoology, University of Puerto Rico, San Juan, Puerto Rico; 7 Department of Physiology, University of Puerto Rico, San Juan, Puerto Rico; 8 Department of Chemistry, University of Puerto Rico, San Juan, Puerto Rico

## Abstract

Antiretroviral therapy partially restores the immune system and markedly increases life expectancy of HIV-infected patients. However, antiretroviral therapy does not restore full health. These patients suffer from poorly understood chronic inflammation that causes a number of AIDS and non-AIDS complications. Here we show that chronic inflammation in HIV+ patients may be due to the disruption of the cholinergic anti-inflammatory pathway by HIV envelope protein gp120_IIIB_. Our results demonstrate that HIV gp120_IIIB_ induces α7 nicotinic acetylcholine receptor (α7) upregulation and a paradoxical proinflammatory phenotype in macrophages, as activation of the upregulated α7 is no longer capable of inhibiting the release of proinflammatory cytokines. Our results demonstrate that disruption of the cholinergic-mediated anti-inflammatory response can result from an HIV protein. Collectively, these findings suggest that HIV tampering with a natural strategy to control inflammation could contribute to a crucial, unresolved problem of HIV infection: chronic inflammation.

Inflammation is a formidable response against pathogens; however, HIV-infected subjects suffer from chronic and persistent inflammatory processes^1, 2, 3^ that promote ‘immunosenescence'^4^ and aging, and trigger AIDS- and non-AIDS-related complications such as neurocognitive deterioration, cardiovascular disease, thromboembolic disease, type 2 diabetes, cancer, osteoporosis, multiple end-organ disease, and frailty.^1, 5, 6^ Inflammation persists indefinitely in HIV+ subjects despite combined antiretroviral treatment, undetectable levels of viremia and even the absence of symptoms.^7, 8^ It has been shown that soluble gp120 contributes to HIV-1 replication and dissemination, via the activation of multiple cell signaling pathways and its presence is associated with higher levels of proinflammatory cytokines in patients.^9^ The latter highlights the need for better understanding of gp120 effects on immune cells to develop new intervention strategies to reduce inflammation and decrease morbidity and mortality in HIV+ individuals.^1, 2^

The cholinergic anti-inflammatory pathway (CAP) modulates the immune response and the progression of inflammatory diseases avoiding organ and systemic damage by inhibiting the release of cytokines.^10^ Although the importance of the CAP in several disease states has been recently established,^11, 12, 13^ the CAP has not been investigated in the inflammatory scenario of HIV infection. Several lines of evidence suggest that the cholinergic anti-inflammatory response (dependent on vagus nerve integrity) could be compromised by HIV infection because infected subjects exhibit hyperactivity of the sympathetic autonomic nervous system or reduction in parasympathetic activity, both at rest and during postexercise recovery,^14^ and autonomic dysfunction is also common in HIV-infected patients being associated with serious comorbid illnesses known to increase mortality risk.^15, 16^ The α7 nicotinic acetylcholine receptor (α7) is a homooligomeric nicotinic acetylcholine (ACh) receptor that is abundantly expressed in the central nervous system. The α7 is characterized by its fast desensitization and high calcium permeability. It is involved in learning and memory, and implicated in neurological disorders such as Parkinson's disease, Alzheimer's disease and schizophrenia. The α7 is also expressed in cells from the immune system such as lymphocytes, monocytes and macrophages.^17, 18, 19^ This transmembrane pentameric ion channel has a pivotal role in the CAP operation because activation of α7 expressed by macrophages inhibits the production of proinflammatory cytokines.^18^ Under basal conditions, the α7 responds to its endogenous agonist ACh by undergoing a conformational change that opens its highly selective calcium-permeable pore. The mechanism by which activation of α7 in macrophages regulates proinflammatory responses is subject of intense research, and important insights have thus been made. The available results suggest that activation of the macrophage α7 controls inflammation by inhibiting nuclear factor-κB nuclear translocation, and activating the JAK2/STAT3 (Janus kinase 2/signal transducer and activator of transcription-3) pathway^20^ among other suggested pathways.^21^ For a comprehensive review of the CAP signaling refer to Báez-Pagán *et al.*^21^ Considering the anti-inflammatory role of α7 activation in macrophages and because HIV+ patients are chronically inflamed, we set out to study this receptor and the cholinergic anti-inflammatory response in the HIV scenario.

Reports suggest that gp120 binds acetylcholine receptors and interferes with cholinergic neurotransmission;^22, 23^ therefore, we rationalized that gp120 could also affect the cholinergic anti-inflammatory response as inflammatory mediators correlate with gp120 levels despite viral suppression by antiretroviral therapy.^24^ Moreover, during chronic HIV-1 infection, a long-term persistence of disproportionately high levels of gp120 have been detected in the absence of virus replication in patients under antiretroviral therapy.^24, 25^ In addition, the presence of anti-gp120 antibodies during chronic, but not acute HIV, infection^26^ demonstrates the importance of studying the cholinergic anti-inflammatory response in chronic HIV-infected patients and the usefulness of studying gp120. Particularly, we focused our efforts in determining the role of clinically relevant doses of gp120_IIIB_ (a CXCR4 tropic-specific gp120) in the chronic inflammation suffered by HIV-infected subjects.

Here we report that HIV-infected subjects are upregulated for α7 in a variety of their immune cells, a phenomena recapitulated by gp120_IIIB_ exposure in monocyte-derived macrophages. Moreover, our results indicate that gp120_IIIB_ disrupts the cholinergic anti-inflammatory response in macrophages because the activation of α7 does not inhibit the production of proinflammatory cytokines (interleukins (ILs) and chemokines). Our findings position α7 as an attractive therapeutic target for the development of novel anti-inflammatory strategies to counteract the chronic inflammation suffered by HIV-infected patients.

## Results

### HIV-1 gp120_IIIB_ induces the upregulation of α7 in monocyte-derived macrophages

**Figure 1:**
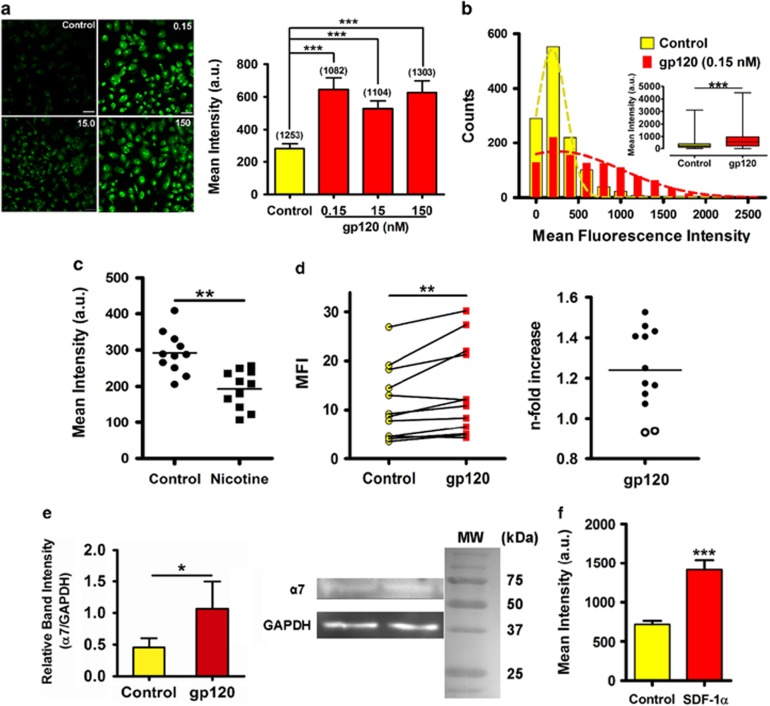
gp120_IIIB_ upregulates the α7 in MDMs isolated from control subjects. (**a**) Confocal imaging revealed that gp120_IIIB_, at various concentrations, increases the α-BuTX binding in MDMs. Scale bar: 50 μm. The total of MDMs analyzed is within parenthesis. ***P<0.0001. Error bars represents s.e.m. (**b**) A frequency histogram analysis shows that a pathophysiological concentration of gp120_IIIB_ produces a right shift toward high mean fluorescent intensity values (*n*=1253 MDMs for control and 1082 for gp120_IIIB_). ***P<0.0001 (inset). Error bars in the inset are box and whisker ranges. (**c**) Nicotine outcompetes α-BuTX binding in MDMs. Nicotine pretreatment followed by α-BuTX addition demonstrates the α-BuTX selectivity for α7 in MDMs. Student's *t*-test, *n*=4 subjects. **P=0.0042. (**d**) Twelve donors were evaluated for α7 levels after gp120_IIIB_ (0.15 nm) exposure. Events were recorded for each donor before and after gp120_IIIB_ treatment. Median fluorescence intensity (MFI) measurements show a significant (**P=0.0034) increase in α7 expression. An *n*-fold representation of the α7 upregulation in these donors show a homogenous population (*n*=12). Open circles represent the only two donors that exhibited a reduction in α7 expression when exposed to gp120_IIIB_. (**e**) MDMs treated with a pathophysiological concentration of gp120 (0.15 nM) exhibit higher α7 protein levels relative to their untreated counterparts (*n*=6). *P=0.0313. (**f**) The endogenous agonist of CXCR4, stromal-derived factor 1α (SDF-1α) (0.3 μg ml^−1^), induces the upregulation of α7. ***P=0.0006. Error bars represent s.e.m. (*n*=3 donors). Statistical analysis for panels **a**, **b** and **f** consists of a paired Student's *t*-test, and for panels **d** and **e**, it consists of a Wilcoxon's signed-rank test; *n*=4 donors for panels **a**–**c**. GAPDH, glyceraldehyde 3-phosphate dehydrogenase.

We performed binding assays using the selective antagonist α-bungarotoxin (α-BuTX) to measure surface α7 protein levels in gp120-treated monocyte-derived macrophages (MDMs) from control subjects (Supplementary Table 1). α-BuTX irreversibly binds the α7 with high affinity (94 pm)^27^ and it is particularly selective for α7.^28^ Figure 1a shows that, following exposure to gp120_IIIB_, there was a significant increase in bound α-BuTX in MDMs from healthy donors, as demonstrated by the shift towards higher fluorescence values in the frequency distribution histogram (Figure 1b). Similar results were obtained using gp120_IIIB_ from the NIH AIDS Reagent Program (data not shown). Consistent with previous studies,^18^ α-BuTX selectively binds α7 in MDMs as demonstrated by the reduced fluorescence intensity upon nicotine pretreatment (Figure 1c). Furthermore, these results were confirmed in a greater number of cells by flow cytometric analysis (Supplementary Figure S1), showing a significant increase in α-BuTX binding in 85% of the examined donors (Figure 1d). This increase was homogeneous among donors, with no evidence of donor sub-populations. Similarly, immunoblot assays showed increased levels of α7 in gp120_IIIB_-treated MDMs (Figure 1e). Furthermore, application of the CXCR4 endogenous agonist stromal-derived factor 1α also induced the upregulation of α7 in MDMs (Figure 1f). Taken together, these results suggest that gp120_IIIB_ induces the upregulation of α7 in human MDMs.

### α7 Nicotinic ACh receptor is upregulated in immune cells from HIV-infected subjects

**Figure 2:**
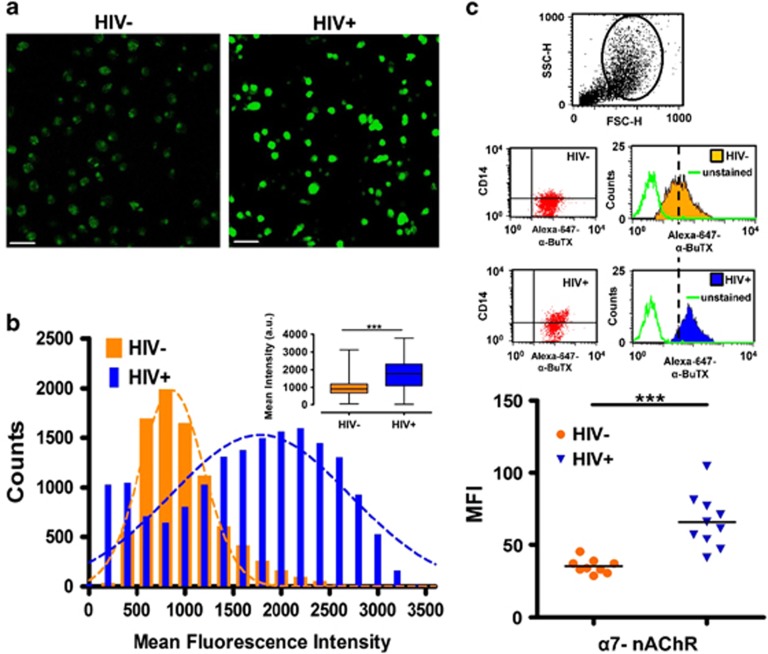
MDMs from HIV+ subjects are upregulated for α7. (**a** and **b**) Confocal imaging using α-BuTX shows significantly (***P<0.0001, inset) higher levels of α7 in HIV+ subjects (*n*=11) relative to HIV− subjects (*n*=12). Scale bar: 50 μm. (**c**) MDMs from HIV− and HIV+ subjects were analyzed using flow cytometry and a significant increase (***P=0.0004; 87%) of α7 was observed in HIV+ relative to HIV−, as determined by the Mann–Whitney *t*-test (HIV− median fluorescence intensity (MFI)=35.3±4.9 vs 66.1±5.9 for HIV− mean±s.d.). Statistical analysis for panel b (inset) consists of a Student's *t*-test and error bars in box and whiskers represent ranges. nAChR, nicotinic acetylcholine receptor.

**Figure 3:**
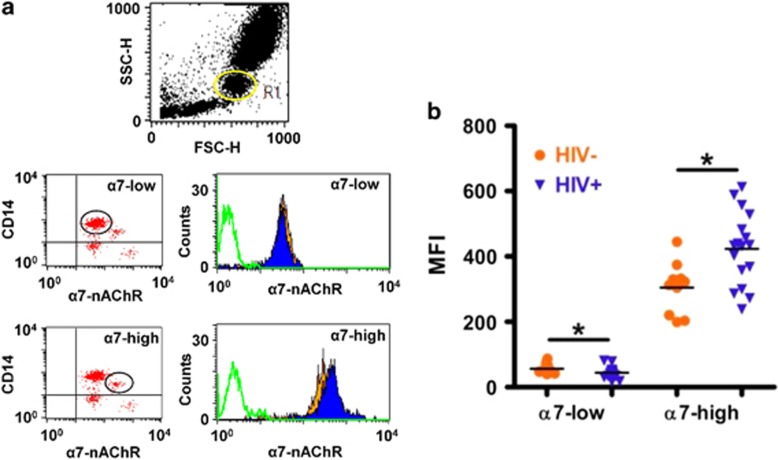
The α7 is upregulated in monocytes from HIV+ subjects. (**a**) Whole-blood analysis from HIV+ and HIV− subjects generated typical scatter plots. Anti-CD14-labeled monocytes were identified and gated (R1) in side scatter (SSC-H)/forward scatter (FSC-H) dot plot. Expression of high α7 levels in CD14^+^ monocytes were analyzed by two-color immunofluorescence as FL1 (CD14^+^)/FL4 (α7^+^) dot plots and histograms. Two populations of monocytes expressing α7 were identified based on Alexa-647-α-BuTX-binding capacity and gated as α7-low and α7-high in the FL1/FL4 dot plots, and analyzed by their corresponding histograms. Open green histograms represent the unstained control monocytes, orange and blue-filled histograms represent HIV− and HIV+ subjects, respectively. (**b**) A significant increase (*P=0.0171; 32%) of α7 expression was detected in the α7-high population (median fluorescence intensity (MFI)=304.8±78.0 for HIV− vs 403.0±110.6 HIV+ mean±s.d.). In addition, we detected a marginal decrease in α7 expression in the α7-low HIV+ cell population that was statistically significant according to the Mann–Whitney test; *n*=10 for HIV− subjects and *n*=17 for HIV+ subjects. nAChR, nicotinic acetylcholine receptor.

**Figure 4:**
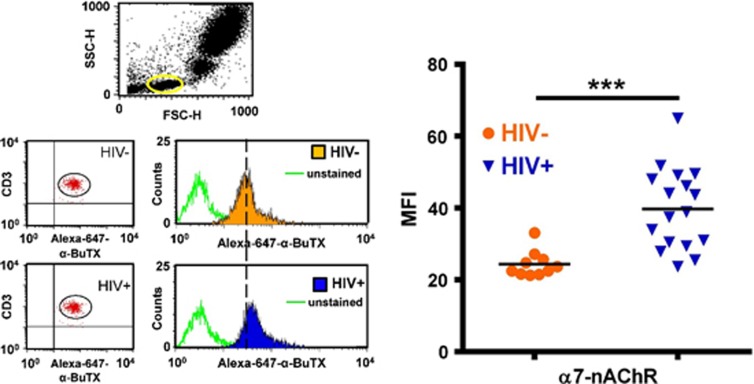
T-lymphocytes from HIV+ subjects are upregulated for α7. T-lymphocytes labeled with anti-CD3 antibody and Alexa-647-α-BuTX were identified and gated (R1) in side scatter (SSC-H)/forward scatter (FSC-H) dot plot. α7 levels in CD3^+^ cells were detected by α-BuTX in two-color immunofluorescence and defined as FL1 (CD3^+^)/FL4 (α7^+^) dot plots. The blue histogram reflects a right shift in cells from HIV+ subjects. Open green histograms represent the unstained cells. In the right panel, a significant (***P=0.0002; 63%) increase of α7 expression was observed in T-lymphocytes from HIV+ subjects (median fluorescence intensity (MFI)=39.8±11.2) relative to HIV− subjects (MFI=24.4±3.63; mean±s.d.). HIV− subjects, *n*=10; HIV+ subjects, *n*=17. The broken vertical line was incorporated in the HIV− histogram peak to compare T-lymphocytes α-BuTX-binding capacity relative to HIV+. Statistical analysis consists of a Mann–Whitney *t*-test. nAChR, nicotinic acetylcholine receptor.

In view of these findings, we asked whether α7 upregulation would also be present in HIV+ individuals. To this end, we measured the α7 levels in samples obtained from HIV+ donors (Supplementary Table 2). Consistent with the aforementioned imaging and flow cytometry results of MDMs exposed to gp120_IIIB_, higher levels of α-BuTx binding were detected by confocal microscopy in the MDMs from HIV+ patients demonstrating that the α7 indeed is upregulated in these subjects (Figures 2a and b). Interestingly, detailed observation of MDMs from controls (Figure 2a) show discrete clusters of α-BuTX binding on the surface consistent with previous work.^18^ To confirm and expand these imaging observations, we analyzed MDMs and other α7-containing immune cells from HIV+ subjects using flow cytometry. We found that the α7 is upregulated in MDMs (Figure 2c), monocytes (Figures 3a and b) and T-lymphocytes (Figure 4) from HIV+ subjects. Interestingly, this approach revealed two distinct populations within monocytes that express low (α7-low) and high (α7-high) levels of α7 (Figures 3a and b), and a substantial increase of α7 in the α7-high cells in HIV+ subjects (Figure 3b). A marginal decrease in α7 expression within the α7-low cells was also observed (Figure 3b). These data indicate that HIV+ subjects exhibit elevated levels of α7 in MDMs, monocytes and T-lymphocytes.

### HIV-1 gp120_IIIB_ disrupts the cholinergic anti-inflammatory response

**Figure 5:**
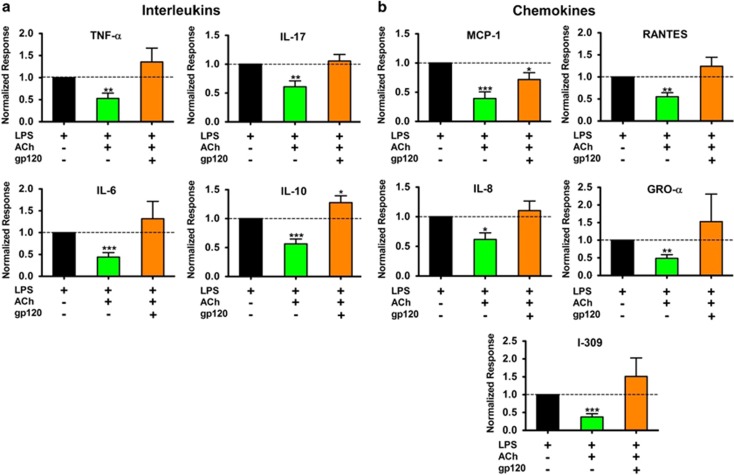
gp120_IIIB_ disrupts the cholinergic anti-inflammatory response of MDMs from uninfected donors. (**a**) IL quantification reveals that, consistent with the CAP operation, ACh addition significantly decreased proinflammatory cytokines (green bar).^18^ However, gp120_IIIB_ pre-exposure (α7 upregulation) abolished the ACh-mediated anti-inflammatory response (orange bar). In agreement with early HIV studies, IL-10 levels increased in MDMs pre-exposed to gp120_IIIB_ as occurs in patients.^63^ However, IL-10 levels were reduced in the presence of ACh. (**b**) Chemokine quantification revealed CAP functioning only in MCP-1 but not in the other chemokines. As occurs with the ILs, the CAP disruption is observed in the majority of chemokines measured. Results were normalized to LPS-induced cytokine release. Normalized response equals the cytokine concentration in the presence of LPS plus ACh divided by the concentration reached with LPS alone ((LPS+ACh/LPS); green bar), and the cytokine concentration of MDMs upregulated for α7 in the presence of LPS plus ACh divided by the concentration reached by LPS alone ((LPS+ACh/LPS); orange bar). Statistical analysis used for both panels: one-sample *t*-test; *n*=8–12 subjects; *P<0.05, **P<0.01 and ***P<0.001.

Given the essential role of α7 in regulating inflammation, we initially hypothesized that high levels of α7 should potentiate the anti-inflammatory response. To test our hypothesis, we measured the secretion of cytokines (ILs and chemokines) in MDMs challenged with lipopolysaccharide (LPS). As expected, ACh reduced the production of proinflammatory cytokines in LPS-treated MDMs.^18^ However, paradoxically, ACh did not reduce the production of cytokines in LPS-treated MDMs previously exposed to gp120_IIIB_ despite the upregulation of the α7 (Figures 5a and b). Furthermore, gp120_IIIB_ did not potentiate LPS-induced release of ILs or chemokines (data not shown). Taken together, these data suggest that gp120_IIIB_, in MDMs, affects the cholinergic anti-inflammatory response, thus disrupting an innate immune response mechanism that controls inflammation.

### An α7 antagonist, bupropion, selectively restores the chemokine-dependent cholinergic anti-inflammatory response

**Figure 6:**
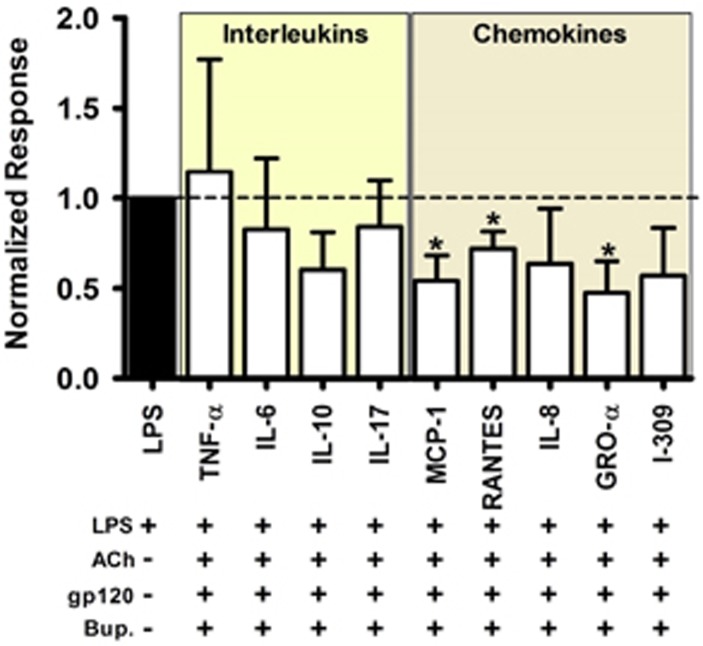
Bupropion (Bup.) reduces proinflammatory chemokines but not ILs in MDMs upregulated for α7. Bup. application did not affect elevated IL levels in MDMs; however, the concentrations of three of the chemokines (MCP-1, RANTES and GRO-α) studied were significantly reduced upon ACh plus Bup. treatment. In addition, Bup. did not affect anti-inflammatory levels of IL-10. Results were normalized to LPS-induced cytokine release. Normalized response equals the cytokine concentration in α7-upregulated MDMs in the presence of Bup., LPS plus ACh and divided by the concentration reached with LPS alone ((Bup.+LPS+ACh/LPS)). Statistical analysis was carried out using one-sample *t*-test; *n*=8–12 subjects. *P<0.05.

We tested whether the medication bupropion (Bup), based on its α7 antagonism together with its well-established clinical use and safety in HIV field,^29, 30, 31^ could restore the anti-inflammatory response in gp120_IIIB_-treated MDMs. We found that Bup selectively restores the CAP in terms of the chemokines GRO-α (growth-related oncogene-α), MCP-1 (monocyte chemoattractant protein-1) and RANTES (regulated on activation, normal T-cell expressed and secreted), but did not have a significant effect on IL-8 and I-309, nor on ILs (Figure 6). These results highlight the potential of α7 targeting to mitigate inflammation in HIV scenarios.

## Discussion

**Figure 7:**
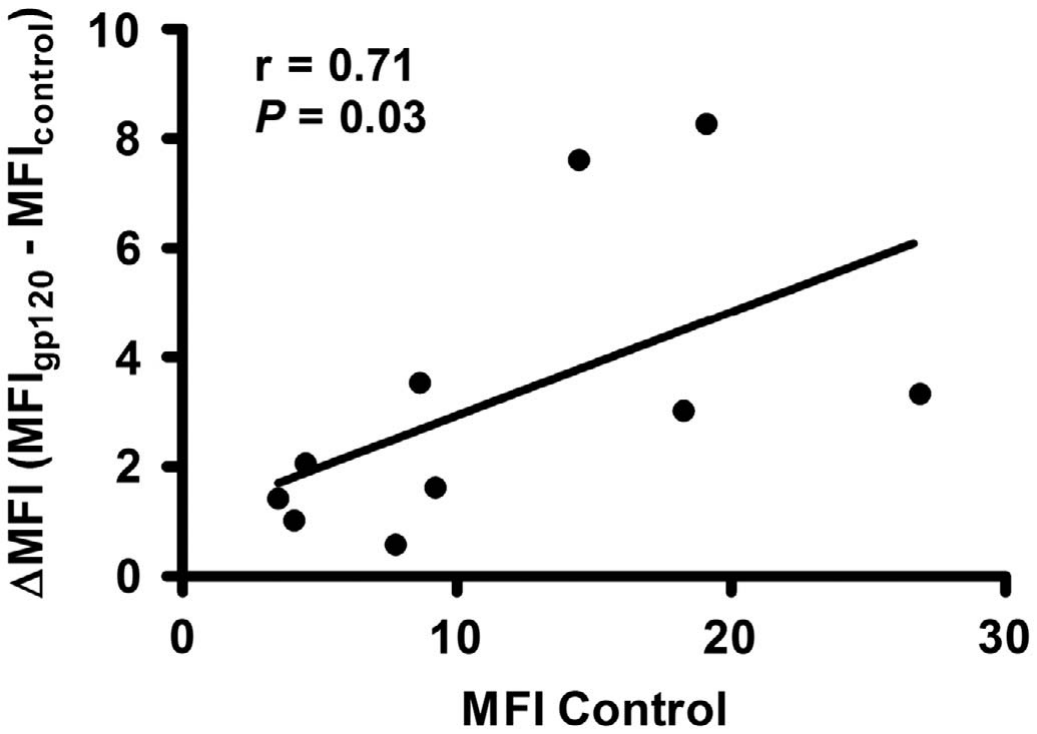
gp120_IIIB_-induced upregulation depends on α7 basal levels in MDMs. We evaluated whether a correlation exists between α7 basal levels and the upregulation observed in uninfected donors. A positive correlation was detected. This correlation was computed by subtracting the gp120 median fluorescence intensity (MFI) values from the control MFI for each donor (ΔMFI). Analysis was conducted by graphing gp120 MFI−control MFI vs control MFI. The correlation analysis was performed using a Spearman's test and a standard linear regression was applied. Correlation was considered significant when *r*>0.3 and *P*<0.05, *n*=10 subjects. For donors' information see Supplementary Table 1.

HIV infection is associated with chronic and persistent inflammation. In this context, inflammation leads to the emergence of a wide spectrum of complications that further compromise patients' health. Moreover, it appears that innate immune responses such as the CAP (dependent on vagus nerve integrity) are also compromised in HIV+ subjects as evidenced by hyperactivity of the sympathetic autonomic nervous system or reduction in parasympathetic activity, both at rest and during postexercise recovery.^14^ Also, combined antiretroviral treatment-independent^32^ alterations in autonomic function have been reported.^33, 34^ Two recent studies began to shed light on the possible role of α7 in different scenarios of HIV pathogenesis. The first report shows that gp120_IIIB_ is able to upregulate α7 in human neuronal cells and the brains of mice expressing gp120_IIIB_ in the central nervous system.^35^ The second report presents evidence that HIV-1 gp120 induces mucus formation in normal human bronchial epithelial cells through a CXCR4-dependent pathway that involve α7 (α7-GABA_A_Rα2).^36^ In our study, we found that a soluble constituent of the HIV-1, gp120_IIIB_, induces the upregulation of α7 in macrophages, as in neuronal cells,^35^ demonstrating the ability of gp120_IIIB_ to upregulate α7 not only in the central nervous system but also in the immune system (Figures 1a, b, d and e). Interestingly, we also identified variations in basal α7 expression levels among donors consistent with previous observations.^37^ Moreover, the extent of the α7 upregulation in MDMs was directly proportional to the basal α7 expression levels (Figure 7). This variation in α7 expression in MDMs is in line with the functional and biochemical heterogeneity of macrophages among subjects^38, 39^ and the differences in their response^40^ that have been proposed to arise from genetic variations. Furthermore, the activation of elevated levels of this highly calcium-permeable channel (α7) did not result in a significant increase in macrophage apoptosis (data not shown), which is consistent with the antiapoptotic signature expressed by monocytes recovered from HIV-infected patients^41^ and macrophages infected with HIV-1.^42^ Interestingly, in HIV+ and HIV− donors we identified two distinctive sub-populations of CD14^+^ monocytes expressing different levels of α7. The origin of these subsets is uncertain. However, we speculate that these differences in α7 expression in monocytes perhaps arise from monocytes' intrinsic genetic heterogeneity (‘classical' and ‘nonclassical' monocytes),^43^ or changes in α7 expression levels during the monocyte/macrophage conversion phase as occurs with other cholinergic receptors.^18^ In the case of HIV-infected patients, particularly, another possibility is that the inflammatory environment present in these patients selectively alters the appropriate assembly of α7 in one population of monocytes over the other, as demonstrated in other cholinergic receptors under proinflammatory settings,^44^ thus disrupting the α-BuTX-binding capacity. Moreover, there is evidence demonstrating that HIV-1 modify monocyte's plasma membrane proteome,^45^ which could also selectively affect the expression of α7 in a specific sub-population of monocytes.

From the experimental side, we asked where do these cells (‘classical' and ‘nonclassical' monocytes) sit in the gating strategy? Our gating strategy only shows the two subsets of monocytes based on CD14/α7 expression. However, we were able to analyze the distinctive pattern distribution of monocyte subsets based on CD14/CD16 expression in a separate experiment. Two sub-populations were identified: the so-called ‘classical' CD14^+^/CD16^−^ monocytes and the ‘nonclassical' CD14low/CD16^+^ monocytes. The position of these two monocyte subsets in the CD14 axis (FL1 channel) was the same as the position of CD14/α7 monocyte subsets. Based on this observation, we hypothesize that α7-high monocytes are the nonclassical CD16^+^ sub-population, whereas α7-low are the classical CD16^−^ subset. This observation is important as both monocyte subsets differ in migration and functionality in HIV infection.^46^ However, these experiments were carried out separately and further studies using a three-color staining protocol for CD14/CD16/α7 is needed to validate this hypothesis. With lymphocytes as well, we observed an increase in α7 levels in CD3^+^ cells from HIV-seropositive patients. We understand that this change may reflect an increase in the receptor expression of both helper (CD4^+^) and cytotoxic (CD8^+^) T-lymphocytes or only in one of these two sub-populations. Thus, the change may reflect different T-lymphocyte sub-populations that become more prevalent in HIV-seropositive patients. Further studies should attempt to quantify α7 levels on different T lymphocytic sub-populations using a four-color panel design of CD3/CD4/CD8/α7 for flow cytometry.

Although we cannot rule out the possibility that other viral proteins are having an important role in the α7 upregulation, we observed that HIV-infected subjects express elevated levels of α7 in their immune cells, a phenomena recapitulated by gp120_IIIB_ addition to MDMs *in vitro*. Paradoxically, the activation of α7-upregulated macrophages did not inhibit the production of inflammatory cytokines and chemokines (Figures 5a and b). These findings highlight a possible viral strategy to disrupt an important innate immune response that neutralizes exaggerated inflammation and thus shed light on the chronic inflammation observed in HIV+ patients.

Our upregulation findings can be discussed from both the viral or host point of view. From the viral point of view, whether the α7 upregulation is beneficial or detrimental to HIV remains unknown. However, the fact that the α7 is highly selective for calcium and the role that calcium has in the transcription,^47^ replication and pathogenesis of HIV^48^ invites the possibility that the virus could modulate α7 expression to allow the necessary calcium influx for its own benefit.^47^ Alternatively, from the host's perspective, it is possible that α7 upregulation represents a frustrated attempt to control inflammation. The observed increase in α7 levels is in accordance with the α7 upregulation reported under inflammation settings in T-lymphocytes, alveolar macrophages and neutrophils.^49, 50, 51^ However, whether this new pool of α7s retains its ligand gated ion channel activity or participate from the anti-inflammatory response remains unknown. Another possibility that we cannot exclude is that the increased expression of α7 in MDMs exposed to gp120_IIIB_ results from endocytosis of neighbor α7s.

**Figure 8:**
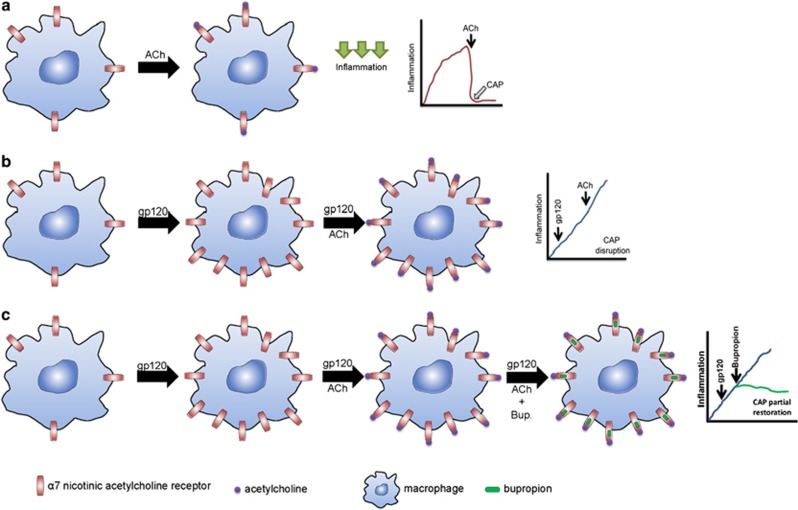
Normal response, disruption and partial restoration of the cholinergic anti-inflammatory response. (**a**) The cholinergic anti-inflammatory pathway promotes homeostasis by decreasing inflammation through the release of ACh and subsequent interaction with the macrophages' α7. (**b**) gp120_IIIB_ exposure promotes α7 upregulation in macrophages and ACh addition does not reduce inflammation. (**c**) Bupropion partially restores the cholinergic anti-inflammatory response by reducing some chemokines but not ILs.

With the advent of combined antiretroviral treatment, the nature of HIV disease has largely shifted from one of immunodeficiency to one of chronic and persistent inflammation and it is recognized that both phenomena are tightly linked in both untreated and treated disease.^1^ In the current study, we conclude that gp120 interferes with the cholinergic anti-inflammatory response because we found that α7 activation is no longer able to reduce the production of proinflammatory ILs and some chemokines (Figures 6 and 8). This result was puzzling because the activation of high levels of α7 was hypothesized to accentuate the anti-inflammatory response in MDMs. Remarkably, however, these results are actually in agreement with the elevated levels of cytokines reported in HIV-infected patients despite the α7 upregulation in MDMs (Figures 2a and b). The ILs that are commonly elevated in patients include tumor necrosis factor-α, IL-6 and IL-17, as well as chemokines, MCP-1, RANTES, IL-8, GRO-α and I-309. Interestingly, although the α7 antagonist tested here, Bup, tends to reduce chemokine production in upregulated macrophages (Figure 6), it has also been shown to reduce proinflammatory ILs in uninfected humans^52^ and experimental animals,^53^ suggesting that, *in vitro*, gp120_IIIB_ interferes with the anti-inflammatory properties of Bup and underscores the complexity of the problem and the need for anti-inflammatory medication tailored to HIV+ patients.

Our observations are significant because they reveal a previously unrecognized alteration in the cells that actively participate in immune response and inflammation. In HIV infection, deregulation of the cytokine networks promotes persistent and chronic inflammation, generating AIDS- and non-AIDS-related complications.^1, 7^ The elucidation of the processes by which HIV/gp120 disrupts the CAP is critical to the development of effective therapeutic strategies aimed at reducing HIV-related chronic inflammation. For instance, the underlying mechanism of the CAP has been suggested to include inhibition of the JAK2/STAT3 pathway, which comprises recruitment of JAK2 to the α7, autophosphorylation of JAK2, phosphorylation of STAT3 by JAK2, dimerization of phosphorylated STAT3 and nuclear translocation of dimerized STAT3 where it exerts its anti-inflammatory role.^20^ The HIV gp120_IIIB_ protein could thus disrupt the CAP by indirectly interfering with JAK2 recruitment to the α7, JAK2 autophosphorylation, the phosphorylation of STAT3 or the nuclear translocation of dimerized STAT3, among other possibilities. In fact, a recent work proposed that gp120 signaling through STAT3 may explain the impairment of dendritic cells upon HIV exposure.^54^

The current study is limited in that the cohort of HIV-infected patients consists of women exclusively. Nevertheless, the reason for using females in our study is that we have access to an extraordinarily well-characterized cohort of HIV+ female patients established by Dr Valerie Wojna. Several publications attest to the scrupulous characterization of this cohort for the past 14 years.^55, 56^

Overall, our findings demonstrate that gp120_IIIB_ can alter the normal function of an innate immune mechanism that controls inflammation and Bup was able to partially rescue it (Figure 6). Moreover, these findings pave the way to study R5-tropic gp120 to determine whether CCR5 stimulation also influences α7 expression levels in MDMs. The present results position the α7 as an attractive therapeutic target that could be exploited as adjunctive therapy to counteract the chronic inflammation that causes a number of AIDS and non-AIDS complications in HIV-infected individuals.

## Methods

### Reagents

All reagents were purchased from Sigma-Aldrich (St Louis, MO, USA), unless otherwise specified.

### Study subjects

All donors enrolled in this study signed the informed consent approved by the Institutional Committee for the Protection of Human Participants in Research (IRB number: 00000944). All experiments were performed in accordance with University of Puerto Rico guidelines and regulations. Phlebotomy to obtain peripheral blood mononuclear cells was performed on uninfected volunteer donors bled at the University of Puerto Rico, Río Piedras for the studies depicted in Figures 1, 5 and 7. Donors were bled at the Puerto Rico Clinical and Translational Research Consortium. All HIV-infected donors were recruited as part of the Hispanic-Latino Longitudinal Cohort of HIV-seropositive women established at the NeuroAIDS Program of the University of Puerto Rico, Medical Sciences Campus. Inclusion criteria included HIV-infected individuals who presented with a CD4 nadir of ⩽500 cells per mm^3^ and/or ⩾1000 copies of plasma viral load while using antiretroviral therapy upon study entry. Women with a history of neuropsychiatric disorders, active infectious process or active drug abuse were excluded. Evaluation consisted of history, neurological exam and neuropsychological test. Smoking history was obtained using the Fagerström Test for Nicotine Dependency Questionnaire.^57^ For detailed HIV+ donor's description, refer to Supplementary Table 2. The peripheral blood mononuclear cells from these HIV− and HIV+ subjects were used for the studies depicted in Figures 2, 3, 4.

### Cell culture

Whole blood from all subjects was processed as described elsewhere.^58^ Peripheral blood mononuclear cells were counted by hemocytometer or Countess automates cell counter (Invitrogen, Eugene, OR, USA), adjusted to 1 × 10^6^ cells per ml and seeded into 75 cm^2^ flasks (Nunc, Rochester, NY, USA) for flow cytometry assays. For confocal imaging of HIV+ and HIV− subjects, 1–2 × 10^6^ cells per ml were cultured into four-well Lab-Tek II Chambered Coverglass (Nalgene, Rochester, NY, USA) as described previously.^58^ For western blot, MDMs were cultured in cell culture Petri dishes (Fisher Scientific, Pittsburgh, PA, USA). After separation of monocytes from lymphocytes by adherence, cells were differentiated for 7–8 days in RPMI-1640 supplemented with 20% inactivated fetal bovine serum, 10% inactivated human serum, 2 μg ml^−1^ macrophage colony-stimulating factor (Invitrogen) and 1% PenStrep. All cultures were maintained at 37 °C with 5% CO_2_. All experiments were performed with cells cultured from a single donor; blood or cells from different donors were not pooled. Cultures, buffers and reagents were endotoxin-free and experiments were performed under aseptic techniques, which included incubators and biological routine monitoring of safety cabinets for microbial growth. Also, the gp120_IIIB_ manufacturer certified that endotoxin levels were ⩽100 EU mg^−1^.

### Western blot

For the western blot, MDM lysates were obtained with lysis buffer (mercaptoethanol diluted in phosphate-buffered saline (PBS) (1 × ) to a final concentration of 2.5%, and supplemented with a protease inhibitor cocktail (Thermo Scientific, Waltham, MA USA; pH 7.4). Protein sample quantification was performed using a Nanodrop (Thermo Scientific). Total homogenate samples, 50 μg, were loaded onto a 10% polyacrylamide gel and run for ~1 h at 30 V, and then at 90 V until completion. After electrophoresis, gels were transferred to a PVDF membrane (Bio-Rad, Hercules, CA, USA) using a wet system (Bio-Rad) for 1 h at 100 V. After this, membranes were incubated in a blocking solution (5% non-fat dry milk, Tris-buffered saline in Tween-20 (TBS-T, 1 × ) for 1 h at room temperature). Subsequently, primary antibody incubation for α7, diluted 1:200 (cat. no.: H-302; Santa Cruz Biotechnology, Santa Cruz, CA, USA), was performed overnight at 4 °C. After three consecutive washes (5 min each) with TBS-T 1 × , an anti-goat secondary antibody labeled with horse peroxidase conjugated and diluted 1:2000 (cat. no.: AP307P; Millipore) was added and incubated for 1 h at room temperature. Membranes were processed using a chemiluminescence assay (Super Signal West Dura Extended Duration Substrate; Thermo Scientific) following the manufacturer's instructions. Blots' relative intensities were evaluated using UVP Vision Works software (UVP, LLC, Upland, CA, USA). The band quantifications are presented as the α7/GAPDH (glyceraldehyde 3-phosphate dehydrogenase) ratio per each experimental condition.

### Confocal imaging

After differentiation, MDMs were incubated and maintained in media supplemented with full-length monomeric glycosylated gp120_IIIB_ expressed in baculovirus, >95% purity by sodium dodecyl sulfate-polyacrylamide gel electrophoresis (Fitzgerald Industries International, Acton, MA, USA), during 72 h or with stromal-derived factor 1α (EMD Chemicals Inc., Gibbstown, NJ, USA) at 0.3 μg ml^−1^. Monomeric gp120 was used for the following reasons: (i) monomeric gp120 interacts with macrophages *in vivo,*^59^ (ii) monomeric and trimeric gp120 induce similar inflammatory responses^59^ and (iii) monomeric gp120 triggers signaling in macrophages similar to those observed with the whole virus.^60^ After incubation, the media were removed and MDMs were washed with PBS 1 × (pH 7.4), followed by fixation with 4% formaldehyde for 15 min at room temperature, washed once with PBS 1 × and labeled with Alexa-488-α-BuTX (Invitrogen) for 1 h at 70 μg ml^−1^ final concentration in the buffer (NaCl, 120 mm; KCl, 4 mm; KH_2_PO_4_, 1.2 mm; MgSO_4_, 1 mm; HEPES, 15 mm (pH 7.4); CaCl_2_, 1 mm; bovine serum albumin, 2% and glucose, 1%). After α-BuTX labeling, MDMs were washed with PBS 1 × to remove unbound α-BuTX, followed by the addition of 90% glycerol/PBS 1 × solution to be finally studied under confocal microscope (Zeiss LSM Meta 510, Carl Zeiss, Pleasanton, CA, USA) at the Confocal Imaging Facility, University of Puerto Rico (http://www.cifupr.org). The remaining bound α-BuTX was excited at a wavelength of 488 nm (0.2%) using an Argon/2 laser and its emission was acquired at 520 nm using a BP 505–550 filter, 64 μm pinhole using a Plan-Apochromat × 20/0.8M27 objective. Images were acquired by random snapshots at 2048 × 2048 dpi followed by background subtractions. Relative fluorescence intensity analyses of each MDM were performed using LSM 510 program. Relative intensities were averaged and plotted. In the case of samples recovered from HIV+ and HIV− subjects, these were prepared as described above, but the incubation time was 30 min. The magnification used for patient samples was × 20 and a 2.0 μm pinhole. The competitive binding assay was performed by adding nicotine to a final concentration of 500 mm before Alexa-488-α-BuTx (2 μg ml^−1^) addition. MDMs were incubated for 15 min at 4 °C in the dark and washed with RPMI-1640 non-supplemented base. Cells were then fixed with 4% formaldehyde-PBS solution (pH 7.2) for 15 min at room temperature. After fixation, MDMs were washed with PBS 1 × (pH 7.2). Finally, Vectashield with DAPI (Vector Labs, Burlingame, CA, USA) was added for visualization and examination by confocal microscopy. Images were collected in Z-stacks at a magnification of × 100 and analyzed. Random snapshots were performed and individual MDMs were analyzed for mean intensity and averaged.

### Flow cytometry

To determine α7 expression levels in monocytes and T-lymphocytes from HIV+ and HIV− donors, freshly drawn blood samples (100 μl) were incubated with the α7 antagonist Alexa-647-α-BuTx (1 h, 4 ºC, 2 μg ml^−1^), CD14-FITC monoclonal antibody (BD Biosciences, San Jose, CA, USA) and CD3-PerCP monoclonal antibody (BD Biosciences), following the manufacturer's instructions. α-BuTx is an α7 antagonist that binds with strong affinity (*K*_d_=94 pm)^27^ and is amply used in α7 expression studies of immune and other cells.^61, 62^ Erythrocytes were lysed by adding 1 × FACS lysis solution (Becton Dickinson, San Jose, CA, USA) for 10 min at 4 °C. Cells were then washed two times with PBS 1 × /fetal bovine serum (3%) by centrifugation at 1100 r.p.m. for 5 min at room temperature. Later, cells were fixed with 0.5% paraformaldehyde and analyzed using flow cytometry. Monocytes were gated in forward scatter (FSC) vs side scatter (SSC) dot plot by size and granularity, and the CD14^+^ and Alexa-647-α-BuTx-labeled cells were identified in FLI vs FL4 dot plot. T-lymphocytes were also gated in FSC vs SSC dot plot, and the CD3^+^ and Alexa-647-α-BuTx-labeled cells were identified in FL3 vs FL4 dot plot. In the case of MDMS, after differentiation, these were labeled with CD14-FITC antibody and Alexa-647-α-BuTx (1 h, 4 ºC, 2 μg ml^−1^). MDMs were gated in FSC vs SSC dot plot, and the CD14^+^ and Alexa-647-α-BuTx-labeled cells were identified in FLI vs FL4 dot plot. For all experiments FITC, PerCP and Alexa-647-α-BuTx emissions were measured in the FL1 (bandpass filter 530/30 nm), FL3 (585/40 nm) and FL4 (bandpass filter 661/16 nm) channels, respectively. Twenty thousand events were analyzed for each sample and the α7 fluorescence intensity of cells was analyzed from the median peak channel of the histograms. Data on scatter parameters and histograms were acquired in log mode. Viability assays for control (97.6%) and gp120_IIIB_-treated MDMs (98.5%) were performed using 7-aminoactinomycin D (BD Biosciences) following the manufacturer's indications and measured in the FL3 channel (585/40 nm). The autofluorescence of monocytes, MDMs and T-lymphocytes was subtracted from the fluorescence intensity values of the stained samples. All samples were assayed using a FACSCalibur (Becton Dickinson) cytometer and analyzed using Cell Quest software (BD Biosciences). This software was used for data acquisition and multivariate analysis.

### IL and chemokine quantification

Peripheral blood mononuclear cells from control subjects were cultured (7–8 days) in 24-well plates, differentiated into MDMs (Supplementary Figure 3A) and assayed for IL and chemokine production after treatments (Supplementary Figure 3B). After differentiation, media were changed for fresh media and gp120_IIIB_ was added for 72 h (to induce α7 upregulation), followed by three consecutive fresh media washes to remove gp120_IIIB_. Later, to test the cholinergic anti-inflammatory response, an inflammation inductor, LPS, was added according to the experimental condition tested. The cholinergic anti-inflammatory response experimental treatments consisted of LPS (100 ng ml^−1^) challenges using *Escherichia coli* O111:B4 (Sigma, St Louis, MO, USA), followed by the addition of ACh (30 μm). The acetylcholinesterase inhibitor pyridostigmine (1 mm) was added 10 min before ACh application to avoid ACh hydrolysis. In the case of Bup (70 ng ml^−1^)-containing assays, to partially antagonize α7, it was added 10 min before LPS or ACh application. Supernatants were collected 20 h post-treatments and stored at −80 °C for further analysis. For further details about experimental design and procedures refer to Supplementary Figures 2 and 3. All supernatants were sent to a contract laboratory (Quansys Biosciences, Logan, UT, USA) for quantification using the multiplex ELISA technology. Samples were analyzed in triplicate.

### Statistical analysis

Nonparametric statistics were used because of the small sample sizes. Comparisons between independent groups were made by using the Mann–Whitney *t*-test, and paired analysis was performed using the Wilcoxon's signed-rank test. One-sample *t*-test was used to compare the gp120 treatment means with LPS (=1). A *P-*value of <0.05 was considered to be significant. The Spearman's test was used to determine associations between two variables, with correlations considered to be significant when *r*>0.3 and *P*<0.05. All statistical analyses were performed with GraphPad (GraphPad, San Diego, CA, USA).
