# Supplementary material for: The α7-nicotinic receptor is upregulated in immune cells from HIV-seropositive women: consequences to the cholinergic anti-inflammatory response
Source: Clin Transl Immunology. 2015 Dec 11;4(12):e53–. doi: 10.1038/cti.2015.31 (PMC4685439; doi:10.1038/cti.2015.31)
Supplement: Supplementary Figure S1 [file cti201531x1.docx]

**
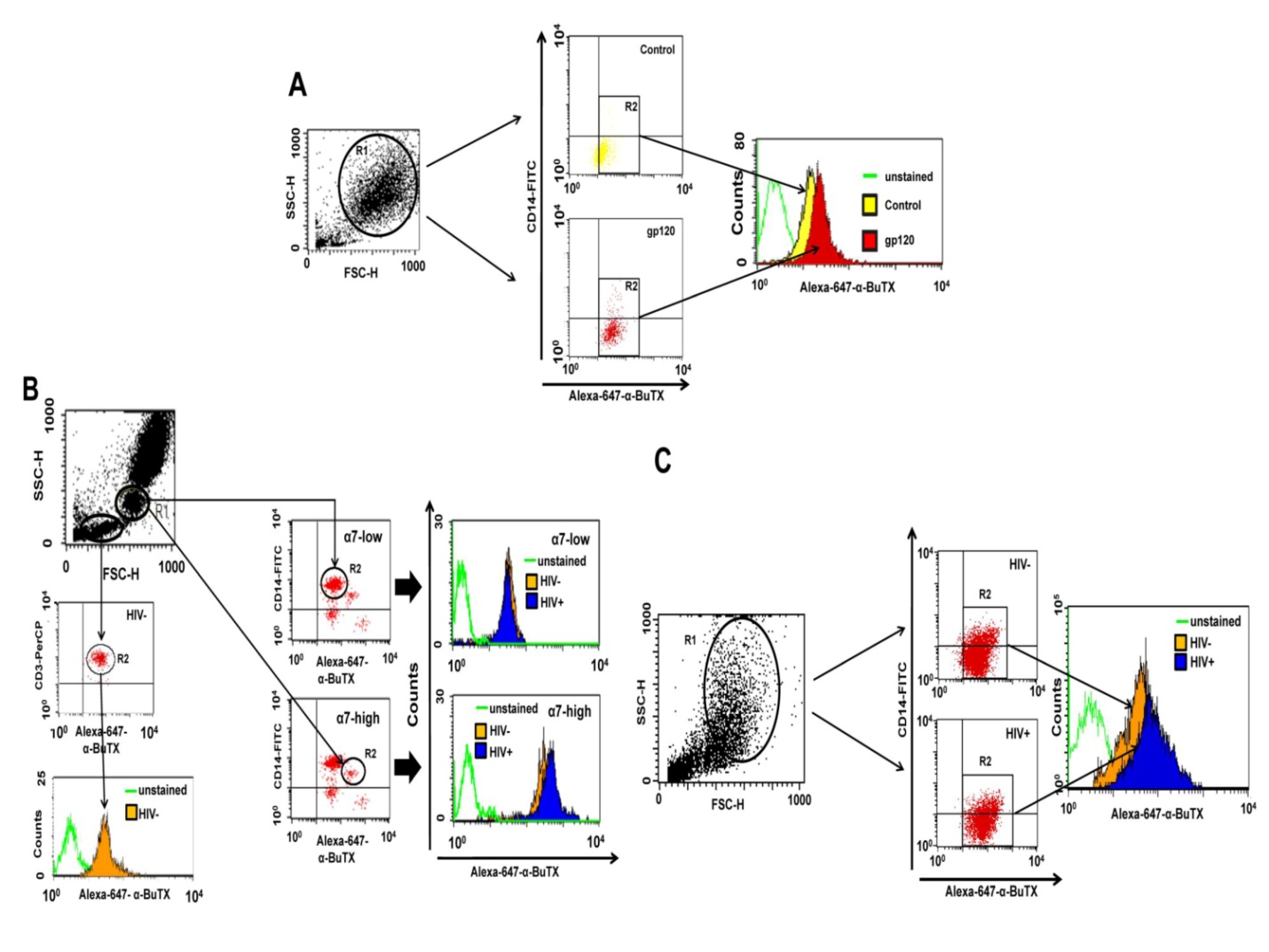
Supplementary Fig. S1.** **Gating strategies used to measure α7 levels in immune cells**. (A) Representative dot plot of gp120-treated and untreated (control) MDMs that were gated (R1) after labeling with CD14-FITC (FL1, SSC) and Alexa-647-α-BuTX for α7 (FL4, FSC). (B) Representative dot plot of whole blood samples in which monocytes (R1) were labeled with CD14-FITC (FL1, SSC) and Alexa-647-α-BuTX for α7 (FL4, FSC). CD14^+^ and α-BuTX^+^ monocytes were gated (R2) and discriminated into two independent populations (α-low and α-high) based on α7 expression levels. The same strategy was used for both HIV- and HIV+ subjects. Also, for T-lymphocytes, whole blood samples were labeled with CD3-PerCP (FL3, SSC) and Alexa-647-α-BuTX for α7 (FL4, FSC). (C) Representative dot plot of MDMs (R1) labeled with CD14-FITC (FL1, SSC) and Alexa-647-α-BuTX for α7 (FL4, FSC). Similar to previous observations, a fraction of MDMs express CD14^51^. MDMs were gated (R2) based on α7 expression levels. In all experiments, horizontal and vertical markers were positioned according to unstained cells that usually were 10^1^.

1. Hopkins, H. A., Monick, M. M. & Hunninghake, G. W. Cytomegalovirus inhibits CD14 expression on human alveolar macrophages. *J. Infect. Dis.* **174,** 69–74 (1996).
