# Supplementary material for: The α7-nicotinic receptor is upregulated in immune cells from HIV-seropositive women: consequences to the cholinergic anti-inflammatory response
Source: Clin Transl Immunology. 2015 Dec 11;4(12):e53–. doi: 10.1038/cti.2015.31 (PMC4685439; doi:10.1038/cti.2015.31)
Supplement: Supplementary Figure S2 [file cti201531x2.docx]

**
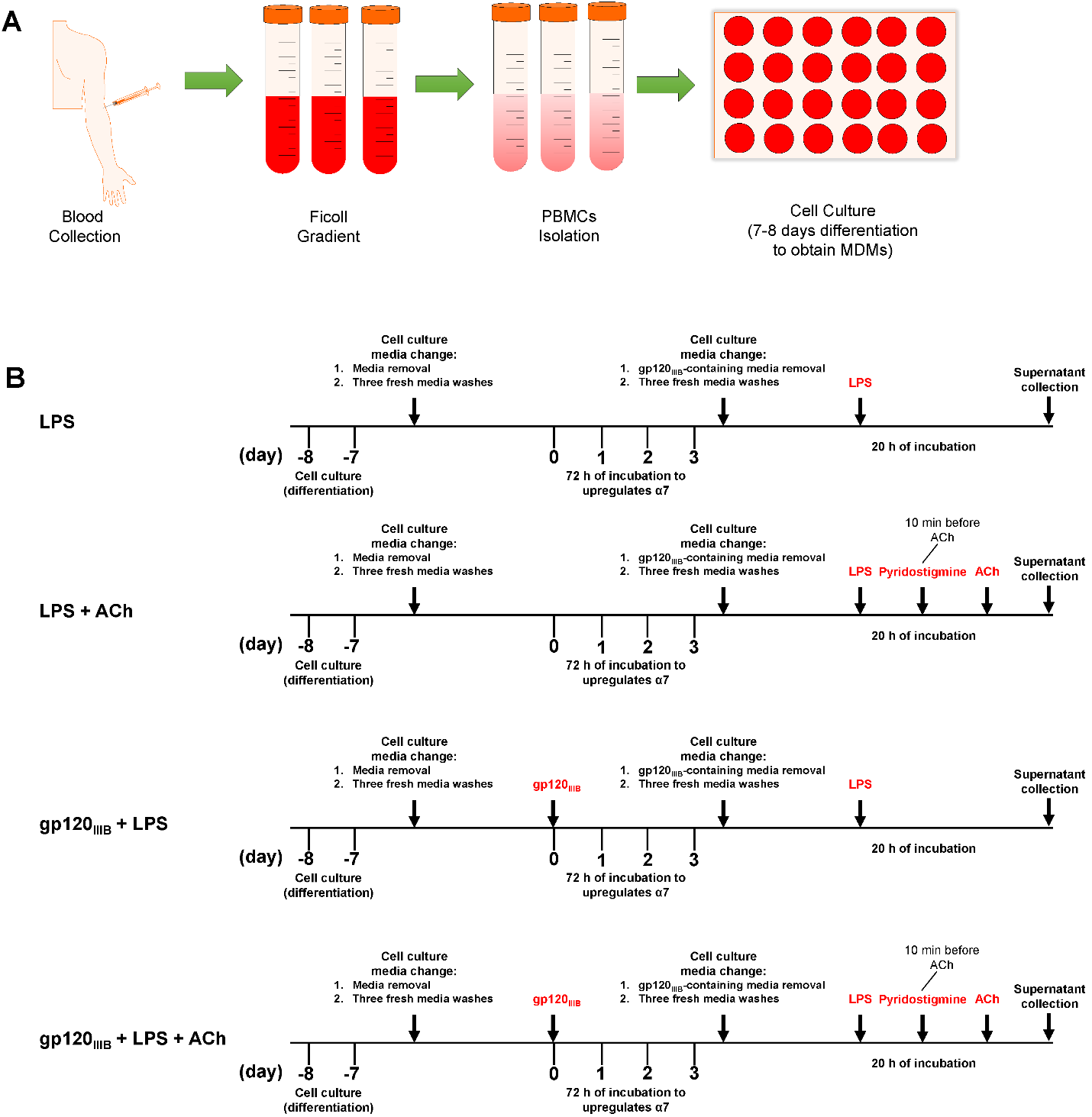
**

**Supplementary Fig. S2.** **Experimental design of cytokines assays**. (A) Blood was collected by venipuncture from healthy donors. After collection it was subjected to Ficoll gradients to obtain PBMCs followed by cell culture to differentiate monocytes into MDMs. (B) Scheme presenting the experimental conditions employed to test the effects of gp120_IIIB_-induced α7 upregulation on the cholinergic anti-inflammatory response in MDMs depicted in Figure 5. LPS was used to induce the production of cytokines in control MDMs, LPS + ACh was used to test the classical cholinergic anti-inflammatory response in control MDMs, gp120_IIIB_ + LPS was used to induce the production of cytokines in MDMs upregulated for α7, gp120_IIIB_ + LPS + ACh was employed to test the cholinergic anti-inflammatory response in MDMs upregulated for α7 by gp120_IIIB_. The acetylcholinesterase inhibitor, pyridostigmine, was always added 10 min before ACh addition to avoid its hydrolysis. After challenging the cholinergic anti-inflammatory response (20 h), supernatants were collected and stored for further cytokines quantification. gp120_IIIB_ was used at 0.15 nM, LPS was used at 100 ng/ml, pyridostigmine was used at 1 mM, and ACh was used at 30 µM. All experiments were performed in parallel.
