# Supplementary material for: The α7-nicotinic receptor is upregulated in immune cells from HIV-seropositive women: consequences to the cholinergic anti-inflammatory response
Source: Clin Transl Immunology. 2015 Dec 11;4(12):e53–. doi: 10.1038/cti.2015.31 (PMC4685439; doi:10.1038/cti.2015.31)
Supplement: Supplementary Figure S3 [file cti201531x3.docx]

**
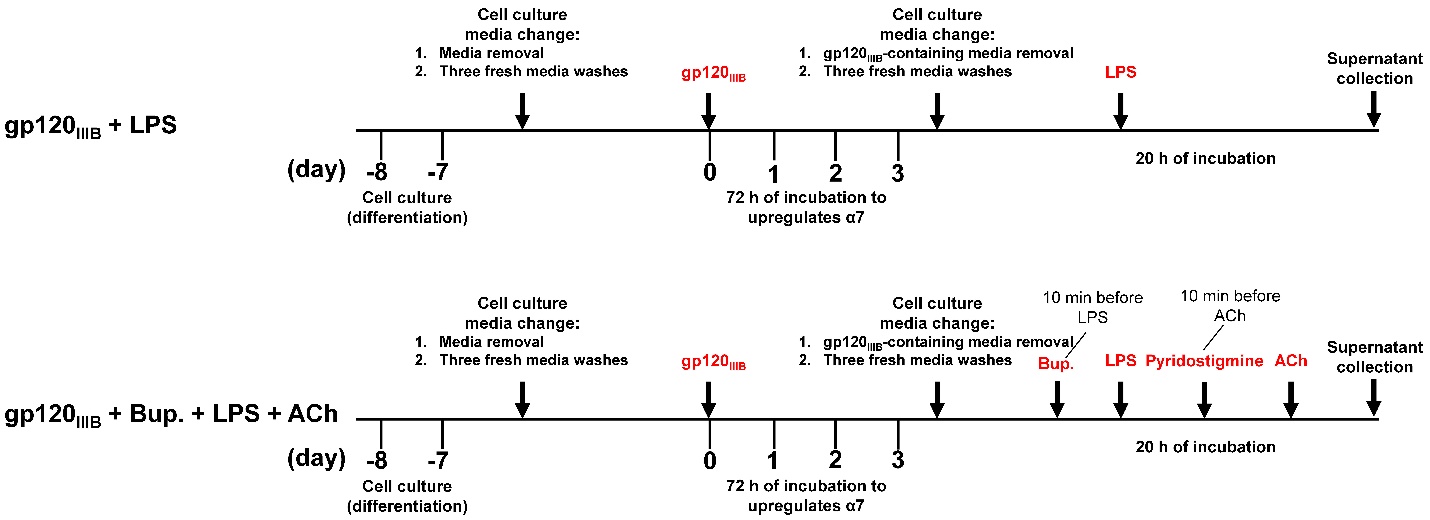
**

**Supplementary Fig. S3.** **Experimental design of cytokines assays upon bupropion treatment in MDMs upregulated for α7**. Scheme presenting the experimental conditions employed to test the effects of gp120_IIIB_-induced α7 upregulation on the cholinergic anti-inflammatory response in MDMs depicted in Figure 6. The partial α7 antagonist bupropion was used in an effort to rescue the cholinergic anti-inflammatory response in upregulated MDMs. LPS was used to induce the production of cytokines in upregulated MDMs, gp120_IIIB_ + Bup. + LPS + ACh was employed to test the cholinergic anti-inflammatory response in MDMs upregulated for α7 by gp120_IIIB_ in the presence of bupropion. After treatments (20 h), supernatants were collected and stored for further cytokines quantification. gp120_IIIB_ was used at 0.15 nM, Bup was used at 70 ng/ml^52,53^, LPS was used at 100 ng/ml, pyridostigmine was used at 1 mM, and ACh was used at 30 µM. All experiments were performed in parallel.

1. [www.fda.gov/AboutFDA/CentersOffices/OfficeofMedicalProductsandTobacco/CDER/ucm153270.htm](http://www.fda.gov/AboutFDA/CentersOffices/OfficeofMedicalProductsandTobacco/CDER/ucm153270.htm)
2. Woodcock J, Khan M, Yu L. Withdrawal of generic budeprion for nonbioequivalence. N Engl J Med 2012; 367: 2463-2465
