# Supplementary material for: The α7-nicotinic receptor is upregulated in immune cells from HIV-seropositive women: consequences to the cholinergic anti-inflammatory response
Source: Clin Transl Immunology. 2015 Dec 11;4(12):e53–. doi: 10.1038/cti.2015.31 (PMC4685439; doi:10.1038/cti.2015.31)
Supplement: Supplementary Table 1 [file cti201531x4.docx]

Supplementary Table 1

|  | **Control Subjects** | |  |
| --- | --- | --- | --- |
| **Variable** | **Male (*n*  = 10)** | **Female (*n* = 10)** | ***^#^P* value** |
| Age  Range | 24.8 (5.8)^*^  19-37 | 27.3 (9.7)^*^ 20-32 | 0.49 |
| Smokers^†^ | 50% | 50% | N/A |

**Supplementary Table 1** General characteristics of control subjects.^*^mean (s.d.); ^†^determined using a questionnaire and interview; value determined using unpaired t-test. Abbreviation: N/A = not applicable.
