# Supplementary material for: The α7-nicotinic receptor is upregulated in immune cells from HIV-seropositive women: consequences to the cholinergic anti-inflammatory response
Source: Clin Transl Immunology. 2015 Dec 11;4(12):e53–. doi: 10.1038/cti.2015.31 (PMC4685439; doi:10.1038/cti.2015.31)
Supplement: Supplementary Table 2 [file cti201531x5.docx]

Supplementary Table 2

|  |  |  |
| --- | --- | --- |
| **Variable** | **HIV+**  **(*n* = 17)** | **HIV- (*n* = 10)** |
| Age  Range | 41.7 (6.9)^*^  26 – 53 | 33.4 (8)^*^  21 – 47 |
| Smokers^†^ | 50% | 50% |
| CD4 cell count (cells/mm^3^)  Range | 478.82 (271.57)^*^  44 – 953 | ND |
| Plasma HIV RNA (Log_10_)^‡,§^  Range | 2.50 (1.06)^*^  1.70 - 4.89  9/17 had no detectable viral load | ND |
| Treatment | 82.4% cART^ǁ^ (14/17)  5.9% ART^¶^ (1/17)  17.6% No treatment (3/17) | N/A |

**Supplementary Table 2** General characteristics of HIV- and HIV+ subjects.^*^mean (s.d.); ^†^determined using the Fagerström Test for Nicotine Dependency; ^‡^ultrasensitive test; lowest copies/ml detectable is 50 copies/ml; ^§^nine of seventeen had no detectable viral load. Abbreviations: ^ǁ^cART = combined antiretroviral therapy (≥ 3 medicaments); ^¶^ART = antiretroviral therapy (≤ 2 medicaments); ND = not determined; N/A = not applicable.
